# *Shigella flexneri *utilize the spectrin cytoskeleton during invasion and comet tail generation

**DOI:** 10.1186/1471-2180-12-36

**Published:** 2012-03-16

**Authors:** Tyson J Ruetz, Ann E Lin, Julian A Guttman

**Affiliations:** 1 Simon Fraser University, Burnaby, B.C. V5A 1S6, Canada; 2 Department of Biological Sciences, Shrum Science Centre, Simon Fraser University, Room B8276, Burnaby, B.C. V5A 1S6, Canada

## Abstract

**Background:**

The spectrin cytoskeleton is emerging as an important host cell target of enteric bacterial pathogens. Recent studies have identified a crucial role for spectrin and its associated proteins during key pathogenic processes of *Listeria monocytogenes *and *Salmonella *Typhimurium infections. Here we investigate the involvement of spectrin cytoskeletal components during the pathogenesis of the invasive pathogen *Shigella flexneri.*

**Results:**

Immunofluorescent microscopy reveals that protein 4.1 (p4.1), but not adducin or spectrin, is robustly recruited to sites of *S. flexneri *membrane ruffling during epithelial cell invasion. Through siRNA-mediated knockdowns, we identify an important role for spectrin and the associated proteins adducin and p4.1 during *S. flexneri *invasion. Following internalization, all three proteins are recruited to the internalized bacteria, however upon generation of actin-rich comet tails, we observed spectrin recruitment to those structures in the absence of adducin or p4.1.

**Conclusion:**

These findings highlight the importance of the spectrin cytoskeletal network during *S. flexneri *pathogenesis and further demonstrate that pathogenic events that were once thought to exclusively recruit the actin cytoskeletal system require additional cytoskeletal networks.

## Background

It is estimated that 164.7 million people worldwide are infected with *Shigella *each year, resulting in ~1.1 million deaths [1]. *Shigella flexneri *are gram-negative, facultative intracellular anaerobic pathogens that can cause full-blown infections from the ingestion of as few as 100 bacteria [2]. These infections trigger the disease shigellosis, characterized by severe inflammatory dysentery, accompanied by watery, bloody diarrhea [1]. Upon ingestion, the bacteria travel throughout the intestinal tract to the colon, where they are phagocytosed by antigen sampling M-cells of the intestinal epithelium and then infect host macrophages and dendritic cells [2,3]. Once within their hosts, they initiate host cell death and are released to the surrounding environment to invade the basolateral surface of intestinal epithelial cells [4]. It is within the cytoplasm of these enterocytes that *S. flexneri *actively replicate and then disseminate to neighboring cells [5].

*S. flexneri *invade enterocytes through bacterially-induced actin-based macropinocytosis; a process similar to *Salmonella *Typhimurium invasion, which is generally referred to as a "triggering" mechanism of bacterial entry [4,6]. This is in contrast to the mode of *L. monocytogenes *entry, which exploit clathrin-based "zippering" internalization mechanisms [7,8]. Once internalized, *S. flexneri *quickly disrupts the vacuolar membrane breaking free into the host cell cytosol [5,6], which is unlike *S*. Typhimurium where upon entry they occupy a phagosome within the infected cells [9]. *S*. *flexneri *then express the IcsA (VirG) protein that localizes to one pole of the bacterial outer membrane. IcsA recruits the actin-associated protein N-WASP, initiating actin polymerization at the bacterial membrane [10]. In a similar manner as during *L. monocytogenes *infections, actin recruitment at one pole of *S. flexneri *creates a "comet tail" that propels the bacterium throughout the host cell and into neighboring cells [11]. Although those comet tail strategies are similar, *L. monocytogenes *utilize the bacterial factor ActA to mimic N-WASP and thus directly recruit the ARP2/3 complex to the bacteria without the need of N-WASP itself [12]. Thus, although *S. flexneri *adopt similar pathogenic strategies as other enteric bacterial pathogens, there are distinct differences that occur during *S. flexneri *infections, requiring researchers to investigate these pathogens independently.

The spectrin cytoskeleton lies just beneath the plasma membrane of eukaryotic cells, providing structural support and protein-sorting capabilities to the membrane [13]. The spectrin sub-membranous scaffold is composed of spectrin heterotetramers, which are interlinked by short actin filaments of 14-16 monomers [14]. Spectrin/actin interactions are facilitated by the spectrin-associated proteins adducin and protein 4.1 (p 4.1), which encourage spectrin-actin binding and can simultaneously bind a number of membrane-associated proteins [15-18]. Consequently, adducin and p4.1 enable the proper anchoring and sorting of membrane associated proteins at the plasma membrane in conjunction with the spectrin scaffold [15,19].

The spectrin cytoskeleton has recently been shown to be important for the pathogenesis of the invasive pathogens *S*. Typhimurium and *L. monocytogenes *[20]. Spectrin, adducin and p4.1 in conjunction with actin are recruited to sites of bacterial/host cell invasion as well as to structures generated at various stages of those intracellular infections. Knockdown of spectrin cytoskeletal components demonstrated that they were necessary for both *S*. Typhimurium and *L. monocytogenes *pathogenesis [20]. Based on these findings, we hypothesized that *S. flexneri *might also exploit spectrin cytoskeletal components during their infections of host cells. In this study we examined the involvement of the spectrin cytoskeleton during the invasion of *S. flexneri *into epithelial cells as well as at later time-points, during the formation of comet tails. We demonstrate striking differences in spectrin cytoskeletal involvement in *S. flexneri *pathogenesis as compared to *S*. Typhimurium or *L. monocytogenes*. We show that p4.1, but not spectrin or adducin, is acutely recruited to the ruffles generated during the initial invasion of *S. flexneri *into epithelial cells, despite all three proteins (spectrin, adducin and p4.1) being crucial for efficient invasion when examined using siRNA-based knockdowns of spectrin components. Further studies demonstrated the recruitment of spectrin, adducin and p4.1 to intracellular bacteria, prior to comet tail formation. However, unlike at *L. monocytogenes *comet tails, we show that spectrin is recruited to *S. flexneri *comet tails. These studies demonstrate a novel cytoskeletal system crucial to *S. flexneri *pathogenesis, while also highlighting dramatic differences between the cytoskeletal hijacking strategies of *S. flexneri, S. *Typhimurium and *L. monocytogenes*.

## Results

### Spectrin cytoskeletal proteins are key components to *S*. *flexneri *invasion of epithelial cells

**Figure 1:**
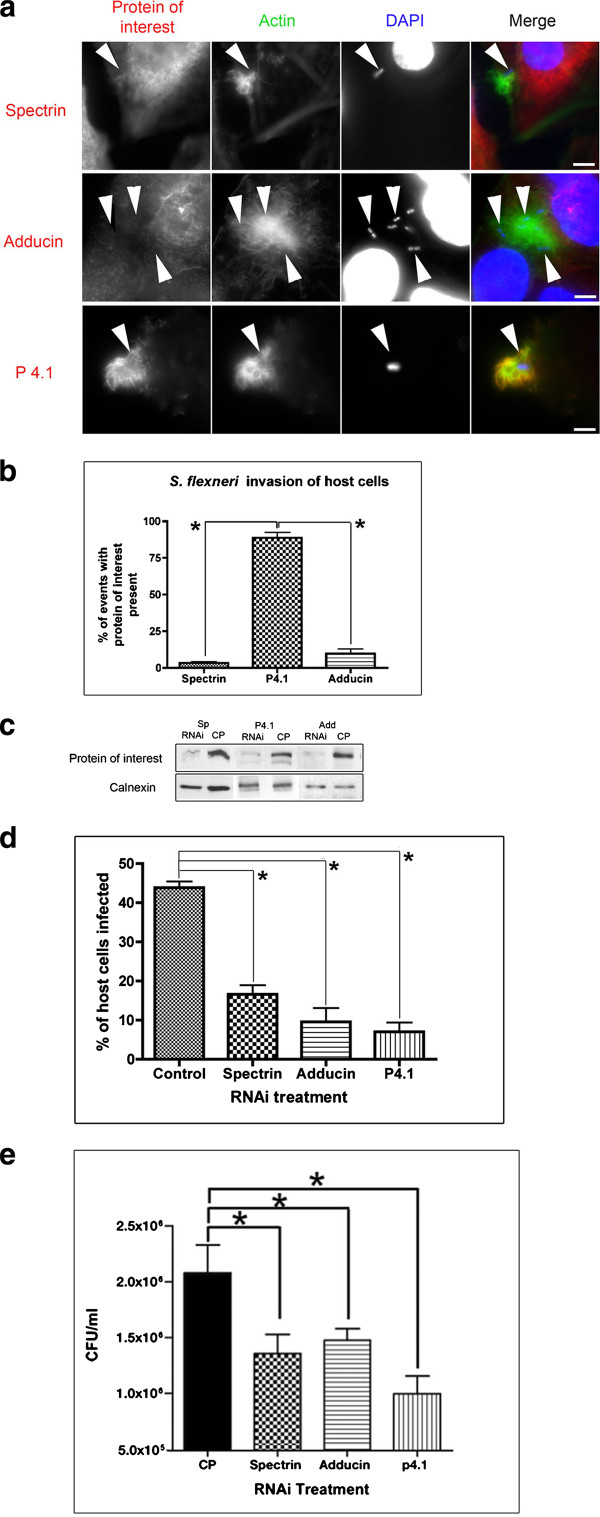
**Spectrin, adducin and p4.1 are needed for efficient *S. flexneri *invasion. a) **HeLa cells were infected with *S. flexneri *for 30 minutes prior to fixation and immunolocalization with antibodies targeted against spectrin, adducin or p4.1. To observe invasion events, we also probed the cells for F-actin (to visualize membrane invasion ruffles) and DNA (using DAPI, to visualize bacteria). P4.1 is recruited to *S. flexneri *actin-rich invasion sites, while spectrin and adducin are not recruited. Scale bars are 5 μm. **b) **Quantification of the presence of spectrin cytoskeletal components during *S. flexneri *invasion. We counted 50 invasion events, in three separate experiements, looking for distinct recruitment of the protein of interest. **c) **Western blots to confirm knockdown of spectrin, adducin and p4.1 in HeLa cells. **d) **Spectrin, adducin, or p4.1 were knocked-down in HeLa cells prior to infection with *S. flexneri *for 1.5 hours (including 1-hour of gentamycin to kill external bacteria), followed by immunolocalization. Quantification of invasion was performed by microscopy, enumerating each cell with 1 or more internalized bacteria as a single invasion event. Cells with spectrin, adducin, or p4.1 knocked-down had significant (*P < 0.0001) reduction in invasion as compared to the control pool treated cells. For each experiment, 25 cells were counted that had undetectable levels of the targeted proteins following knockdown. In the case of control pool treated cells, random fields of view were chosen. Each experiment was run in triplicate. **e) **Classical invasion assay whereby spectrin, adducin, or p4.1 were knocked-down in HeLa cells prior to infection with *S. flexneri *for 30 minutes, followed by 1-hour gentamycin treatment. Cells were lysed and bacteria loads were recovered by CFU enumeration. Cells with protein knock-downs exhibit a significant decrease in *S. flexneri *invasion. Experiments run in triplicate. * p < 0.05

To examine the role of spectrin cytoskeletal proteins during *S. flexneri *invasion, we infected HeLa cells with *S. flexneri *for 30 minutes and immunolocalized spectrin, adducin and p4.1. To identify bacterial sites of invasion, indicated by actin-rich membrane ruffles, we probed the cells with Alexa fluor conjugated phalloidin (to stain filamentous actin) as well as DAPI (to visualize bacterial DNA). We found that p4.1 was recruited to 94% of *S. flexneri *invasion sites (Figure 1a and 1b, Additional file 1: Figure S1 showing background actin). However, spectrin and adducin were largely absent from sites of *S. flexneri *invasion, showing recruitment to only 3% and15% of invasion sites respectively (Figure 1a and 1b).

We then sought to identify if any of the spectrin cytoskeletal proteins influenced *S. flexneri *invasion. To accomplish this, we utilized pools of 4 siRNA's targeted against spectrin, adducin and p4.1 to knockdown those proteins in cells prior to infection with *S. flexneri*. To control for non-specific/off target effects of the siRNA treatments, we transfected cells with a control pool of 4 non-targeting siRNAs [20]. Successful knockdowns were confirmed using western blots (Figure 1c). Actin filaments remain unaltered during spectrin cytoskeletal knockdowns [20]. SiRNA pre-treated cells were infected with *S. flexneri *for 30-minutes, followed by 1-hour gentamycin treatment to kill external bacteria, prior to fixation and subsequent immunolocalization. We then enumerated the total number of cells infected, counting each cell with 1 or more bacterium inside as 1 infection event. We observed a significant reduction in *S. flexneri's *ability to invade cells in the absence of each spectrin cytoskeletal protein. In cells with undetectable levels of spectrin, adducin, or p4.1, we observed 38%/22%/16% invasion (respectively) as compared to *S. flexneri *infections of the control pool (control) treated cells (Figure 1d). The important role for spectrin cytoskeletal components during invasion was confirmed using a classical invasion assay, with gentamycin treatment, showing significant decreases in invasion when any of the spectrin cytoskeletal components were knocked down (Figure 1e). Because siRNA mediated knockdown is not 100% efficient, the classical invasion assay results include cells with incomplete knockdowns, hence the reduction in total invasion is not as dramatic as in Figure 1e compared to 1 d. Microscopic analysis revealed cells with unsuccessful knockdown beside cells with near complete knockdown in the same field of view. This analysis demonstrated bacterial invasion of cells with unsuccessful knockdown and lack of bacteria within the successfully knocked-down cells (Additional file 2: Figure S2).

### Intracellular *S. flexneri *recruits spectrin cytoskeletal proteins at key stages of the infections

**Figure 2:**
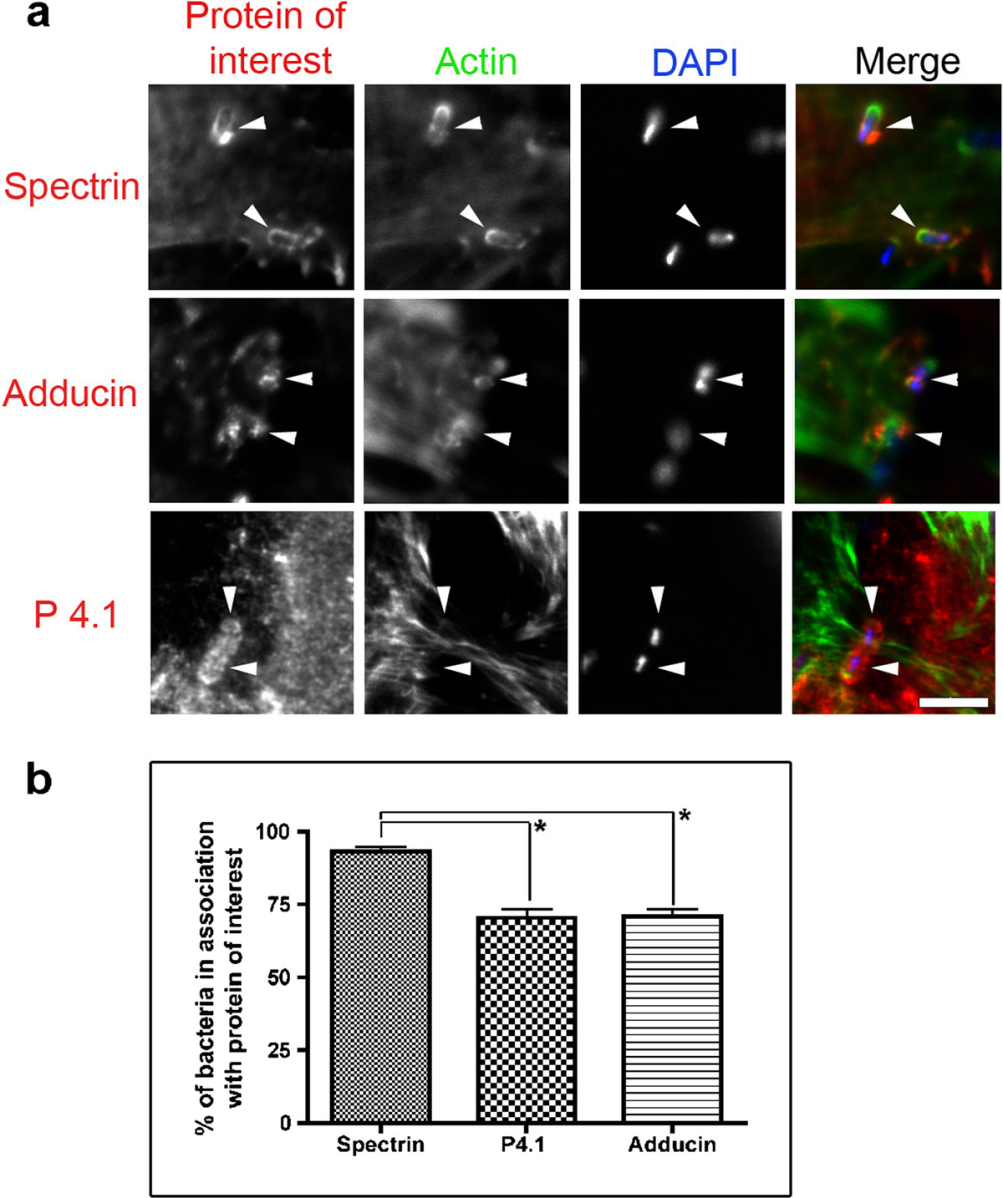
**Spectrin cytoskeletal proteins are recruited to internalized *S. flexneri***. Cells were infected for 2.5 hours prior to fixation and treatment with antibodies targeted to spectrin, adducin or p4.1, together with probes for F-actin and DAPI (to visualize the DNA within the bacteria). **a) **All three proteins are recruited to the regions containing the internalized bacteria (arrowheads). Spectrin and adducin panels show instances where spectrin cytoskeletal proteins were concentrated in the absence of actin. Scale bars are 5 μm. **b) **Quantification of spectrin, p4.1 and adducin recruitment to internalized bacteria. 200 internalized bacteria were counted, in three separate experiments, to observe if they had recruited spectrin cyskeletal proteins to the bacteria. * p < 0.05

To examine the intracellular life of *S. flexneri*, we began by observing internalized bacteria 2.5 hours after the initial infections. At this stage of the infections, the bacteria can replicate within the host cell cytoplasm and some are at the initial phases of recruiting actin to produce the characteristic comet tails. When we examined spectrin, adducin and p4.1, we noticed recruitment of these proteins localized to the area of internalized bacteria (Figure 2a). F-actin only partially co-localized with some of the areas of spectrin cytoskeletal protein recruitment, with many bacteria having only recruited the spectrin cytoskeletal proteins at this stage of the infections (Figure 2a and Additional file 3: Figure S3). We examined the proportion of the bacteria that associated with spectrin cytoskeletal proteins, irrespective of actin recruitment, and found that 95%, 72%, and 73% of internalized bacteria were associated with spectrin, p4.1 and adducin at 2.5 hours post infection (Figure 2b).

**Figure 3:**
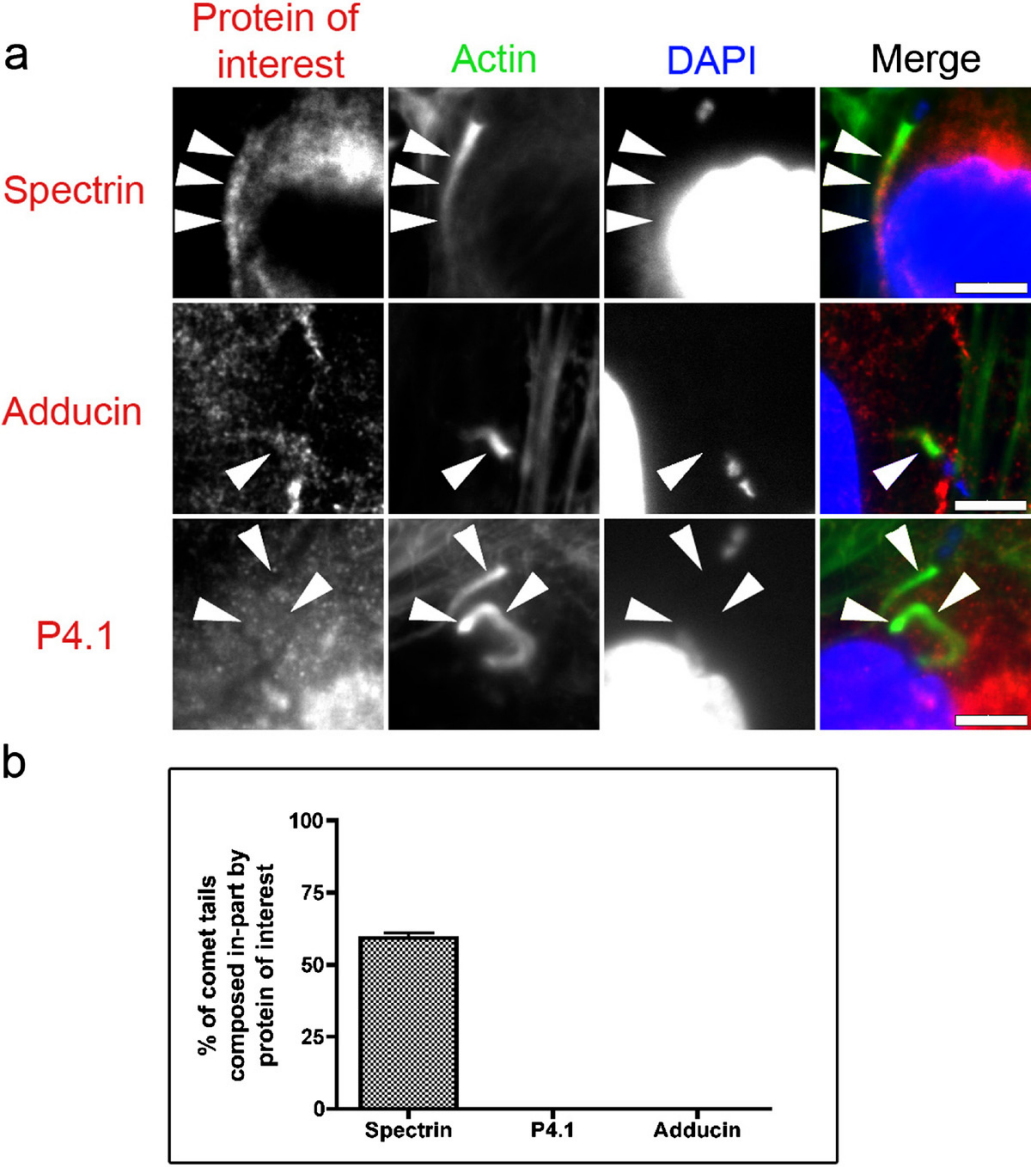
***S. flexneri *recruit spectrin, but not adducin or p4.1 to comet tails**. HeLa cells were infected with *S. flexneri *for 4.5 hours prior to fixation and immunolocalization with antibodies against spectrin, adducin and p4.1. Actin and DNA (DAPI) probes identify comet tails and bacteria respectively. **a)** Spectrin is recruited to *S. flexneri *comet tails, while adducin and p4.1 were absent. Arrows indicate comet tail regions of interest. Scale bars are 5 μm. **b)** Quantification of spectrin, p4.1, or adducin recruitment to *S. flexneri *comet tails. 50 comet tails were counted in three separate experiments to observe if the protein of interest was recruited to the tail. Spectrin was recruited to 61% of tails, while p4.1 and adducin were not observed recruited to tails in any instance.

We then investigated *S. flexneri *during the intracellular motile stage, when the bacteria utilize actin-rich comet tails to propel throughout the host cytoplasm. After 4.5 hours of infection, many of the bacteria have produced the tail structures. We infected cells for 4.5 hours and then visualized the spectrin cytoskeletal proteins in conjunction with F-actin. Spectrin was recruited, albeit not as intensely as actin, to 61% of *S. flexneri *comet tails, colocalizing with actin (Figure 3a and 3b). Specifically, spectrin localization within the comet tail was strongest at regions where F-actin was less abundant, being most intensely found ~2-3 μm distal to the interface between the actin-tail and bacterium (Figure 3a). Adducin and p4.1 were not recruited to the comet tail (Figure 3a and 3b).

## Discussion

Previous studies have shown that the membranous ruffles triggered by *S*. Typhimurium and the actin-rich comet tails generated during *L. monocytogenes *infections share some morphological and structural characteristics with *S. flexneri *during their infectious process [6,21]. *S. *Typhimurium and *L. monocytogenes *recruit and require spectrin cytoskeletal proteins for their efficient invasion as well as for subsequent infectious stages within their host cells [20]. Based on these similarities, we hypothesized that *S. flexneri *may also exploit spectrin cytoskeletal proteins during their infections. Here we have identified important roles for the spectrin cytoskeleton during *S. flexneri *initiated macropinocytic invasion of host cells and their presence at comet tails.

During *S. flexneri *invasion, a multitude of actin cytoskeletal-associated proteins are recruited to membrane ruffles triggered by T3SS translocated bacterial effectors [6]. We found that during *S. flexneri *infections, p4.1 but not spectrin or adducin, localized to 94% of invasion events. Despite the near complete absence of spectrin or adducin recruitment, when any of the three proteins were disrupted through siRNA treatments, invasion of *S. flexneri *was severely decreased. How can the decreased expression of spectrin cytoskeletal proteins that are not markedly recruited to invasion sites have such a dramatic impact on *S. flexneri *invasion? Clues to understanding this can be derived from previous research investigating spectrin cytoskeletal involvement during cell migration. There are many shared protein components and structural similarities between *S. flexneri *membrane invasion ruffles and membrane protrusions generated during cell migration events. During cell migration, spectrin, adducin and p4.1 often co-localize with, and are necessary for, the recruitment and correct localization of actin-associated machineries to the sub-membranous region of the plasma membrane [14,23,23]. Knockdown of p4.1, or functional perturbation of adducin, both result in an inhibition of membrane protrusions and lack of cell motility [22,24]. Thus, it is plausible that proteins involved in actin dynamics leading to the formation of *S. flexneri *membrane ruffles and their subsequent invasion are mis-localized when spectrin, adducin, or p4.1 is knocked down. This could explain the observed decrease in bacterial invasion in their absence. Despite not being intensely localized at sites of invasion, we did observe faint recruitment of spectrin and adducin at these invasion sites.

The lack of robust spectrin and adducin recruitment to *S. flexneri *invasion sites did not parallel what was found once the bacteria had invaded the host cells, as all three spectrin cytoskeletal components were found surrounding internalized bacteria. We observed their recruitment to invaded bacteria, in the absence of actin, suggesting that those proteins likely arrived at the bacterial interface prior to the recruitment of actin and subsequent comet tail formation. It is possible that spectrin and associated proteins may help recruit the actin machinery to the bacteria, similarly to how they function within lamellipodia [22,24], to produce the comet tails enabling intracellular motility. Unfortunately, due to the low abundance of bacteria internalized during spectrin cytoskeletal knockdowns, we were unable to investigate the impact of spectrin cytoskeletal protein involvement in actin recruitment to internalized bacteria.

Upon *S. flexneri *generation of full-length actin-rich comet tails, spectrin was found at the comet tails, while p4.1 and adducin were not. Previous work that decorated filamentous actin with the S1 subfragment of myosin identified *S. flexneri *comet tails to be dense networks of branched and cross-linked actin filaments [21]. Cross-linking proteins, such as α-actinin, are recruited to *S. flexneri *comet tails and are thought to provide the bacteria with a rigid platform off of which they propel [21,25]. Spectrin is an established actin cross-linking protein, increasing the viscosity of actin filaments *in vitro *[26]. This cross-linking characteristic may be at work within *S. flexneri *comet tails, however this requires further scrutiny.

As the actin dynamics at the leading edge of motile cells are similar to those occurring during pathogen induced macropinocytotic membrane ruffling and comet tail motility, one would predict that similar components would be present at these sites. *L. monocytogenes *and *S. flexneri *have been used as model systems to study pseudopodial protrusions for years [27,28]. However, the identification of only spectrin and not adducin or p4.1 at fully formed *S. flexneri *comet tails, together with the absence of all spectrin cytoskeletal components at *L. monocytogenes *comet tails [20], highlight differences between membrane protrusion events during whole cell motility and those generated by bacterial pathogens. These findings demonstrate the diverse tactics used by microbes to regulate host components and further show that pathogens exploit varying factors during their infectious processes.

Our findings, and findings from other papers (summarized in Additional file 4: Table S1) demonstrate that not all components of the spectrin cytoskeleton always act in concert. Rather, we have observed that spectrin, adducin, and p4.1 can act in the absence of each other during the pathogenic processes of *S. flexneri, L. monocytogenes, S*. Typhimurium and Enteropathogenic *E. coli *(EPEC) pathogenesis. Previous studies have highlighted roles for spectrin, adducin and p4.1, irrespective of the influence of one another. Adducin is capable of binding, cross-linking and bundling F-actin, in the absence of spectrin and p4.1 [29]. Similarily, spectrin is capable of binding actin in the absence of adducin or p4.1 [18]. Furthermore, purified spectrin and p4.1 can cross-link actin filaments *in vitro*, in the absence of adducin [26]. Adducin is also capable of binding a number of plasma membrane proteins, in the absence of spectrin [30]. Thus, precedents from other systems support our findings that spectrin, adducin, and p4.1 can act independently during bacterial pathogenesis.

## Conclusions

Invasion of intestinal epithelial cells and comet tail-based motility in host cells are key for *S. flexneri *to access replicative niches and disseminate throughout host tissues [2]. Here we have demonstrated that the actin-rich structures generated by these microbes also employ another cytoskeletal system, the spectrin cytoskeleton. Our identification of this structural network at these sites further highlights the importance of this system in bacterial pathogenesis and indicates that these crucial segments in the pathogenesis of *S. flexneri *require a hybrid cytoskeletal meshwork, previously thought to be exclusive to actin.

## Methods

### Cells, bacteria and growth conditions

HeLa cells (ATCC) were grown on #1 cover slips in Dulbecco's Modified Eagles Medium (DMEM) supplemented with 10% fetal bovine serum (FBS). The bacterial strain utilized was *S. flexneri *(strain M90T). Bacteria were grown in standard trypticase soy.

### Infections

HeLa cells were grown to approximately 70% confluency prior to infections. *S. flexneri *were grown overnight in standing culture, then diluted 80×, followed by growth in shaking culture at 37°C for 2.5 hours (OD600 nm = 0.6) after which 400 μl of the culture was added to the cells with 200 μl of growth media [31]. Infections were initiated by centrifugation for 10 mins at 700 g and 21°C. To quantify invasion events, investigate initial tail formation and study comet tails, total infection times consisted of 0.5, 2.5 and 4.5 hours respectively. For classical invasion assays, cells were washed 2× with PBS after 20 minutes of infections and incubated in 100 ug/mL of gentamycin in 10% DMEM for 1 hour. Cells were washed 3× with PBS, lysed using 1% triton and plated for CFU counts.

### Invasion assays examined by microscopy

To quantify *S. flexneri *invasion, similar infection parameters were followed as in the classical invasion assay, however after 1 hour of gentmycin treatment the cells were washed with PBS three times prior to fixation and quantification of bacterial invasion via microscopy.

### Immunofluorescence

Immunofluorescence procedures were performed as described previously [20]. Briefly, samples were fixed using 3% paraformaldehyde for 15 minutes then permeabilized using 0.1% Triton X-100 in PBS (without calcium or magnesium) (Hyclone) for 5 minutes. Prior to primary antibody treatments, samples were blocked in 5% normal goat serum in TPBS/0.1% BSA (0.05% Tween-20 and 0.1% BSA in PBS) for 20 minutes. Antibodies were then incubated on the cover slips overnight at 4°C. The next day secondary antibodies were applied for 1.5 hrs at 37°C. The cover slips were then mounted on glass slides using Prolong Gold with DAPI (Invitrogen). All micrographs were acquired using a Leica DMI4000B inverted fluorescent microscope equipped with a Hamamatsu Orca R2 CCD camera (Hamamatsu, Japan). Metamorph Imaging System software was used to run the microscope and obtain the images (Universal Imaging Corp., Pennsylvanian).

### Immunolocalization reagents

Primary antibodies consisted of a mouse monoclonal anti-β-Spectrin II (used at 2.5 μg/ml for immunofluorescence, 0.025 μg/ml for westerns) (Becton Dickinson), a rabbit anti-α-adducin (used at 2 μg/ml for immunofluorescence and 0.02 μg/ml for westerns) (Santa Cruz Biotechnology), rabbit anti-EPB41 (protein 4.1) (used at 1.7 μg/ml for immunofluorescence and 0.017 μg/ml for westerns)(Sigma Aldrich), and rabbit anti-calnexin (Becton Dickinson) (used at 1:2000). Secondary antibodies included goat anti-mouse or anti-rabbit antibodies conjugated to AlexaFluor 568 or 594 (used at 0.02 μg/ml) (Invitrogen). For F-actin staining AlexaFluor 488 conjugated phalloidin (Invitrogen) was used at a 1:10 dilution for 7 minutes, according to the manufacturers instructions. DNA was visualized using the mounting medium Prolong Gold with DAPI (Invitrogen).

### Transfection of siRNA and confirmation of knockdowns via western blots

Pools of 4 targeted siRNAs were used simultaneously to independently knockdown β-Spectrin II, protein 4.1, α-adducin [20]. A control pool of 4 non-targeting siRNAs (Dharmacon) was used to control for off target effects. All transfections were performed using the InterferIN transfection reagent (PolyPlus Transfection), over a period of 48 hours, according to the manufactures instructions. The media was changed to standard DMEM with 10% FBS prior to the infections. Western blots were performed to confirm successful knockdown as outlined previously [20].

For assays that used siRNA-treated cells, the coverslips were examined microscopically, initially for cells that had complete knockdown of the protein of interest, then the number of bacteria in the cells were assessed by first confirming the bacteria were inside of the cells by scanning the samples from top to bottom and acquiringZ-stacks.

### Statistics

Statistical analysis involved a 1-way ANOVA analysis, with Dunnett's post-hoc test, to compare each data set to the control group. When we compared data sets directly, we used a non-parametric student t-test.

## Authors' contributions

TJR conceived, designed and performed experiments, analyzed the data and co-wrote the paper. AEL designed and performed invasion assay experiments and analyzed the data. JAG helped design experiments and co-wrote the paper. All authors read and approved the final manuscript.

## Supplementary Material

Additional file 1 **Figure S1 Modified Figure 1 with brightened actin**. A modified version of Figure 1 with the actin levels brightened to show the actin in other regions of the host cell. This figure exemplifies how concentrated actin is at the site of *S. flexneri *infection. Scale bar is 5 μm Click here for file

Additional file 2 **Figure S2 RNAi images of *S. flexneri *infections showing non-transfected cells next to cells with near complete knockdown of spectrin, p4.1, or adducin**. Spectrin, adducin, or p4.1 were knocked-down in HeLa cells prior to infection with *S. flexneri *for 1.5 hours (including 1-hour of gentamycin to kill external bacteria), followed by microscopy analysis. Cells with spectrin cytoskeletal proteins knocked down show the absence of internalized bacteria. Whereas arrows identify neighboring cells in the same field of view with unsuccessful transfection, expressing spectrin cytoskeletal proteins, which have robust infection. Scale bar is 5 μm Click here for file

Additional file 3 **Figure S3 Low magnification images of cells with internalized *S. flexneri***. Cells were infected for 2.5 hours prior to immunofluorescent visualization of spectrin, adducin or p4.1, together with probes for F-actin and DAPI (to visualize the DNA within the bacteria). These images are to support Figure 2 by showing the overall distribution of spectrin cytoskeletal proteins in cells with robust *S. flexneri *infection. Arrows indicate areas of cells with internalized *S. flexneri*, showing the rearrangements of spectrin, adducin or p4.1 in those areas. Scale bar is 5 μm Click here for file

Additional file 4 **Table S1 Summary of spectrin cytoskeletal involvement during various stages of enteric bacterial disease**. Table provides a comprehensive summary of the presence or absence of spectrin, p4.1 and adducin at key stages of *S. flexneri, L. monocytogenes, S. *Typhimurium and EPEC pathogenesis Click here for file

## References

[B1] Peng J Yang J Jin Q The molecular evolutionary history of Shigella spp. and enteroinvasive Escherichia coli Infect Genet Evol 2009 9 147 152 10.1016/j.meegid.2008.10.003 19000785

[B2] Ashida H Ogawa M Mimuro H Sasakawa C Shigella infection of intestinal epithelium and circumvention of the host innate defense system Curr Top Microbiol Immunol 2009 337 231 255 10.1007/978-3-642-01846-6_8 19812985

[B3] Keren DF McDonald RA Wassef JS Armstrong LR Brown JE The enteric immune response to shigella antigens Curr Top Microbiol Immunol 1989 146 213 223 10.1007/978-3-642-74529-4_23 2659270

[B4] Mounier J Vasselon T Hellio R Lesourd M Sansonetti PJ Shigella flexneri enters human colonic Caco-2 epithelial cells through the basolateral pole Infect Immun 1992 60 237 248 1729185 10.1128/iai.60.1.237-248.1992 PMC257528

[B5] Ray K Bobard A Danckaert A Paz-Haftel I Clair C Ehsani S Tang C Sansonetti P Tran GV Enninga J Tracking the dynamic interplay between bacterial and host factors during pathogen-induced vacuole rupture in real time Cell Microbiol 2010 12 545 556 10.1111/j.1462-5822.2010.01428.x 20070313

[B6] Cossart P Sansonetti PJ Bacterial invasion: the paradigms of enteroinvasive pathogens Science 2004 304 242 248 10.1126/science.1090124 15073367

[B7] Veiga E Cossart P Listeria hijacks the clathrin-dependent endocytic machinery to invade mammalian cells Nat Cell Biol 2005 7 894 900 10.1038/ncb1292 16113677

[B8] Veiga E Guttman JA Bonazzi M Boucrot E Toledo-Arana A Lin AE Enninga J Pizarro-Cerda J Finlay BB Kirchhausen T Cossart P Invasive and adherent bacterial pathogens co-Opt host clathrin for infection Cell Host Microbe 2007 2 340 351 10.1016/j.chom.2007.10.001 18005755 PMC2803069

[B9] Kumar Y Valdivia RH Leading a sheltered life: intracellular pathogens and maintenance of vacuolar compartments Cell Host Microbe 2009 5 593 601 10.1016/j.chom.2009.05.014 19527886 PMC2716004

[B10] Suzuki T Miki H Takenawa T Sasakawa C Neural Wiskott-Aldrich syndrome protein is implicated in the actin-based motility of Shigella flexneri EMBO J 1998 17 2767 2776 10.1093/emboj/17.10.2767 9582270 PMC1170617

[B11] Kocks C Marchand JB Gouin E d'Hauteville H Sansonetti PJ Carlier MF Cossart P The unrelated surface proteins ActA of Listeria monocytogenes and IcsA of Shigella flexneri are sufficient to confer actin-based motility on Listeria innocua and Escherichia coli respectively Mol Microbiol 1995 18 413 423 10.1111/j.1365-2958.1995.mmi_18030413.x 8748026

[B12] Boujemaa-Paterski R Gouin E Hansen G Samarin S Le Clainche C Didry D Dehoux P Cossart P Kocks C Carlier MF Pantaloni D Listeria protein ActA mimics WASp family proteins: it activates filament barbed end branching by Arp2/3 complex Biochemistry 2001 40 11390 11404 10.1021/bi010486b 11560487

[B13] Baines AJ Evolution of spectrin function in cytoskeletal and membrane networks Biochem Soc Trans 2009 37 796 803 10.1042/BST0370796 19614597

[B14] Bennett V Baines AJ Spectrin and ankyrin-based pathways: metazoan inventions for integrating cells into tissues Physiol Rev 2001 81 1353 1392 11427698 10.1152/physrev.2001.81.3.1353

[B15] Baines AJ The spectrin-ankyrin-4.1-adducin membrane skeleton: adapting eukaryotic cells to the demands of animal life Protoplasma 2010 244 99 131 10.1007/s00709-010-0181-1 20668894

[B16] Baines AJ Evolution of the spectrin-based membrane skeleton Transfus Clin Biol 2010 17 95 103 10.1016/j.tracli.2010.06.008 20688550

[B17] Li X Matsuoka Y Bennett V Adducin preferentially recruits spectrin to the fast growing ends of actin filaments in a complex requiring the MARCKS-related domain and a newly defined oligomerization domain J Biol Chem 1998 273 19329 19338 10.1074/jbc.273.30.19329 9668123

[B18] Ohanian V Wolfe LC John KM Pinder JC Lux SE Gratzer WB Analysis of the ternary interaction of the red cell membrane skeletal proteins spectrin, actin, and 4.1 Biochemistry 1984 23 4416 4420 10.1021/bi00314a027 6487610

[B19] Beck KA Nelson WJ The spectrin-based membrane skeleton as a membrane protein-sorting machine Am J Physiol 1996 270 C1263 C1270 8967424 10.1152/ajpcell.1996.270.5.C1263

[B20] Ruetz T Cornick S Guttman JA The spectrin cytoskeleton is crucial for adherent and invasive bacterial pathogenesis PLoS One 2011 6 e19940 10.1371/journal.pone.0019940 21603579 PMC3095645

[B21] Gouin E Gantelet H Egile C Lasa I Ohayon H Villiers V Gounon P Sansonetti PJ Cossart P A comparative study of the actin-based motilities of the pathogenic bacteria Listeria monocytogenes, Shigella flexneri and Rickettsia conorii J Cell Sci 1999 112 11 1697 1708 10318762 10.1242/jcs.112.11.1697

[B22] Ruiz-Saenz A Kremer L Alonso MA Millan J Correas I Protein 4.1R regulates cell migration and IQGAP1 recruitment to the leading edge J Cell Sci 2011 124 2529 2538 10.1242/jcs.083634 21750196

[B23] Bournier O Kroviarski Y Rotter B Nicolas G Lecomte MC Dhermy D Spectrin interacts with EVL (Enabled/vasodilator-stimulated phosphoprotein-like protein), a protein involved in actin polymerization Biol Cell 2006 98 279 293 10.1042/BC20050024 16336193

[B24] Fukata Y Oshiro N Kinoshita N Kawano Y Matsuoka Y Bennett V Matsuura Y Kaibuchi K Phosphorylation of adducin by Rho-kinase plays a crucial role in cell motility J Cell Biol 1999 145 347 361 10.1083/jcb.145.2.347 10209029 PMC2133101

[B25] Sanger JW Sanger JM Cell motility. Beads, bacteria and actin Nature 1992 357 442 10.1038/357442a0 1489395

[B26] Fowler V Taylor DL Spectrin plus band 4.1 cross-link actin. Regulation by micromolar calcium J Cell Biol 1980 85 361 376 10.1083/jcb.85.2.361 6892816 PMC2110616

[B27] Cossart P Lecuit M Interactions of Listeria monocytogenes with mammalian cells during entry and actin-based movement: bacterial factors, cellular ligands and signaling EMBO J 1998 17 3797 3806 10.1093/emboj/17.14.3797 9669997 PMC1170715

[B28] Lambrechts A Gevaert K Cossart P Vandekerckhove J Van Troys M Listeria comet tails: the actin-based motility machinery at work Trends Cell Biol 2008 18 220 227 10.1016/j.tcb.2008.03.001 18396046

[B29] Mische SM Mooseker MS Morrow JS Erythrocyte adducin: a calmodulin-regulated actin-bundling protein that stimulates spectrin-actin binding J Cell Biol 1987 105 2837 2845 10.1083/jcb.105.6.2837 3693401 PMC2114693

[B30] Lu Q Liu X Trama J Roti MA Go WY HO SN Identification of the cytoskeletal regulatory protein alpha-adducin as a target of T cell receptor signaling Mol Immunol 2004 41 435 447 15163540 10.1016/j.molimm.2004.03.028

[B31] Mostowy S Bonazzi M Hamon MA Tham TN Mallet A Lelek M Gouin E Demangel C Brosch R Zimmer C Entrapment of intracytosolic bacteria by septin cage-like structures Cell Host Microbe 2010 8 433 444 10.1016/j.chom.2010.10.009 21075354

