# Supplementary figures and images for: Shigella flexneri utilize the spectrin cytoskeleton during invasion and comet tail generation
Source: BMC Microbiol. 2012 Mar 16;12:36. doi: 10.1186/1471-2180-12-36 (PMC3384245; doi:10.1186/1471-2180-12-36)

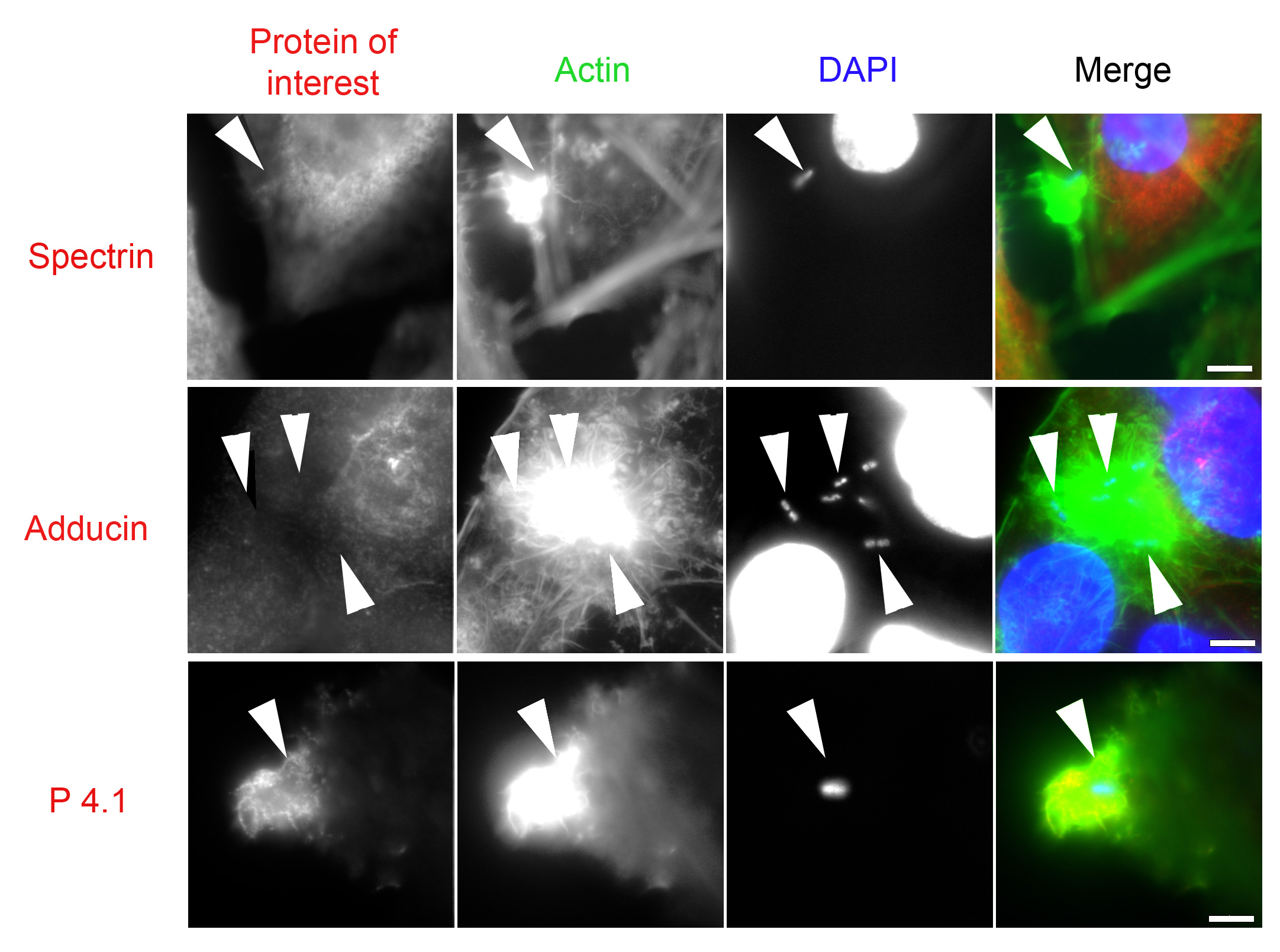

Supplement: Additional file 1 — Figure S1 Modified Figure 1 with brightened actin. A modified version of Figure 1 with the actin levels brightened to show the actin in other regions of the host cell. This figure exemplifies how concentrated actin is at the site of S. flexneri infection. Scale bar is 5 μm [file 1471-2180-12-36-S1.JPEG]

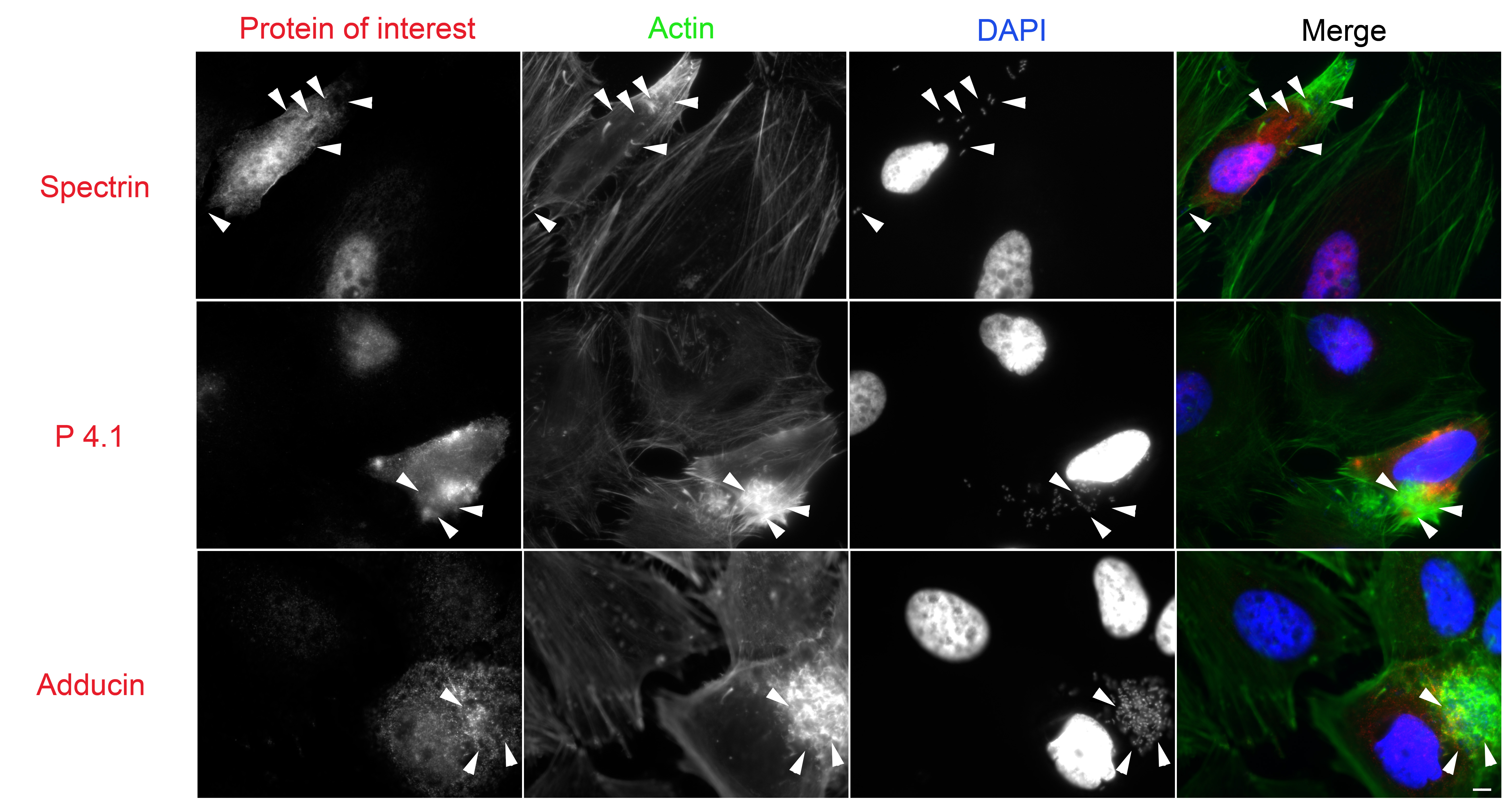

Supplement: Additional file 2 — Figure S2 RNAi images of S. flexneri infections showing non-transfected cells next to cells with near complete knockdown of spectrin, p4.1, or adducin. Spectrin, adducin, or p4.1 were knocked-down in HeLa cells prior to infection with S. flexneri for 1.5 hours (including 1-hour of gentamycin to kill external bacteria), followed by microscopy analysis. Cells with spectrin cytoskeletal proteins knocked down show the absence of internalized bacteria. Whereas arrows identify neighboring cells in the same field of view with unsuccessful transfection, expressing spectrin cytoskeletal proteins, which have robust infection. Scale bar is 5 μm [file 1471-2180-12-36-S2.JPEG]

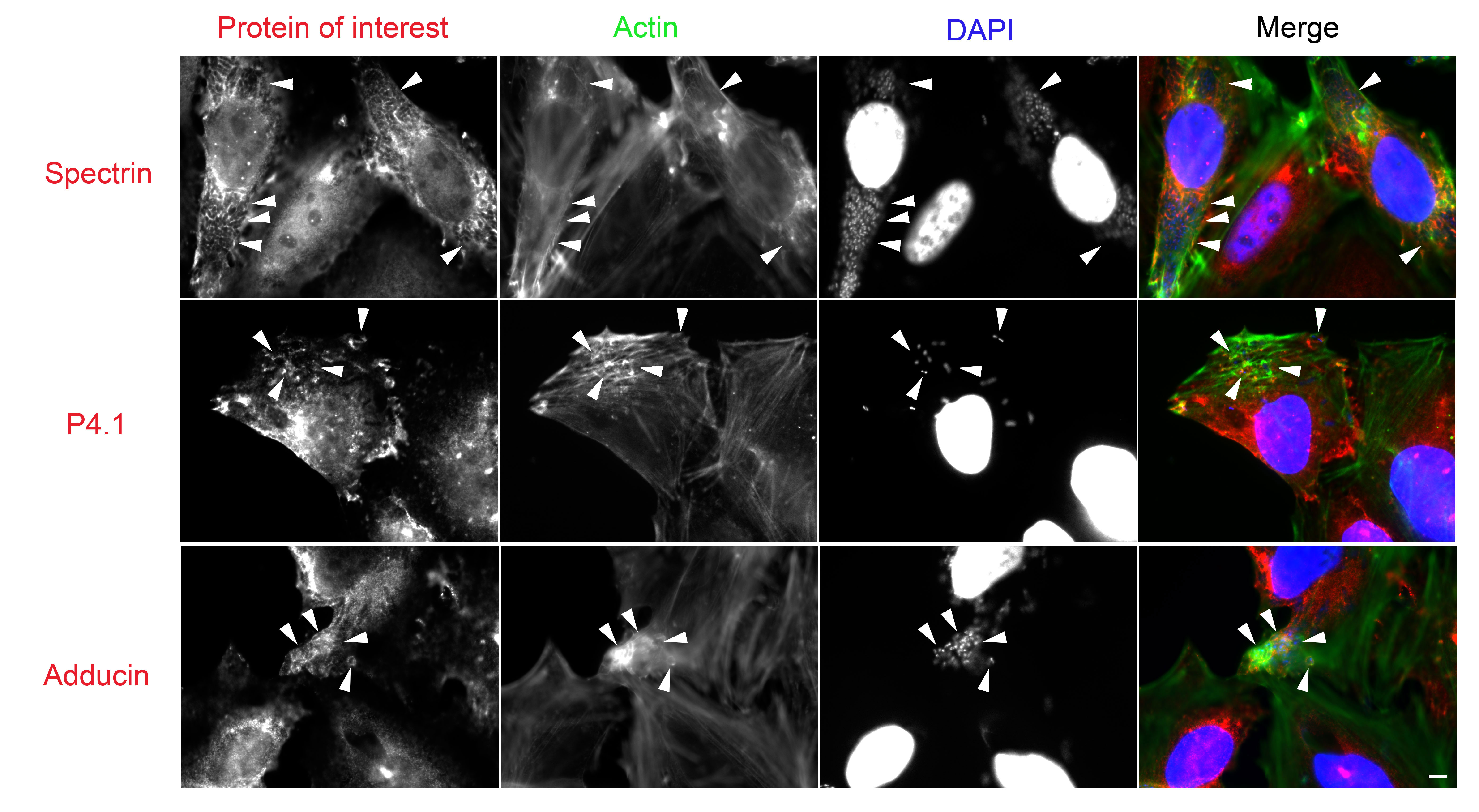

Supplement: Additional file 3 — Figure S3 Low magnification images of cells with internalized S. flexneri. Cells were infected for 2.5 hours prior to immunofluorescent visualization of spectrin, adducin or p4.1, together with probes for F-actin and DAPI (to visualize the DNA within the bacteria). These images are to support Figure 2 by showing the overall distribution of spectrin cytoskeletal proteins in cells with robust S. flexneri infection. Arrows indicate areas of cells with internalized S. flexneri, showing the rearrangements of spectrin, adducin or p4.1 in those areas. Scale bar is 5 μm [file 1471-2180-12-36-S3.JPEG]
