# Supplementary material for: Shigella flexneri utilize the spectrin cytoskeleton during invasion and comet tail generation
Source: BMC Microbiol. 2012 Mar 16;12:36. doi: 10.1186/1471-2180-12-36 (PMC3384245; doi:10.1186/1471-2180-12-36)
Supplement: Additional file 4 — Table S1 Summary of spectrin cytoskeletal involvement during various stages of enteric bacterial disease. Table provides a comprehensive summary of the presence or absence of spectrin, p4.1 and adducin at key stages of S. flexneri, L. monocytogenes, S. Typhimurium and EPEC pathogenesis [file 1471-2180-12-36-S4.PDF]

Pathogen/process	Spectrin	P 4.1	Adducin	References
S. flexneri				
Invasion	-	+	-	This paper
Internalized bacteria	+	+	+	This paper
Comet tails	+	-	-	This paper
S.**Typhimurium**				
Invasion	+	+	+	29
SCV	+	-	-	29
L. monocytogenes				
Invasion	+	+	+	29
Early stage comet tail	+	+	-	29
Comet tail	-	-	-	29
**EPEC**				
Pedestals	+	+	+	29
